# Factors associated with adoption acceptance rate from the view point of infertile couples 

**Published:** 2012-09

**Authors:** Seyyed Mojtaba Yassini, Mohsen Taghavi Shavazi, Naeimeh Taghavi Shavazi

**Affiliations:** 1 *Research and Clinical Center for Infertility, Shahid Sadoughi University of Medical Sciences, Yazd, Iran.*; 2 *Tehran Heart Center, Tehran University of Medical Sciences, Tehran, Iran.*; 3 *Ali Ben Abi Taleb Medical College, Yazd Branch, Islamic Azad University, Yazd, Iran.*

**Keywords:** *Infertility*, *Adoption*, *Artificially assisted reproductive techniques*

## Abstract

**Background:** Nowadays artificially assisted reproductive techniques are used to cure infertility. These methods are highly expensive, time-consuming and have low success rates which are usually around 20-40%. One of the best alternate methods for infertility treatment that can be considered is adoption that often decreases the treatment costs and the psychological impact within an infertile couple.

**Objective:** This study has been done with the aim of determining adoption acceptance rates and the effective factors of adoption in infertile couples.

**Materials and Methods:** A cross-sectional study was performed between October 2009-2010 on 200 infertile couples who had been referred to Infertility Center of Shahid Sadoughi University of Medical Sciences. Information gathered through face-to-face interview and questionnaires. The data analyzed through a SPSS software program using ANOVA test.

**Results:** There was a significant statistical relationship between adoption acceptance value scores and marriage duration of a couple (p=0.002 in men, p=0.004 in women) and presence of adoption backgrounds in male relatives (p=0.004). There was no statistically significant relationship between age, gender, education level, and onus of infertility, the number of previous referrals for an infertility solution and presence of adoption backgrounds in female relatives.

**Conclusion:** Adoption as an alternative option to infertility treatment need to be more considered as a medical, social and cultural issue.

## Introduction

Infertility is one of the most contentious issues in modern society. Infertility means the effective non-occurrence of pregnancy after one year of having sexual intercourse without the use of means to prevent pregnancy (1-5). The rate of infertility in the world is about 10-15% (3-5) and this number exceeds to 20% in Iran according to past research studies (6, 7). Nowadays artificially assisted reproductive techniques are often used to treat infertility (3-5).

However this not only imposes expensive treatment but this is also rather time-consuming and the success rate is about 20-40% (3, 5). Adoption as an alternative process is the method whereby a child is accepted into a family by one or more adult persons (7, 8). The adoptive parents are not the biologic parents but are considered as legal parents instead (7). 

This method can decrease both the treatment costs and the psychological impact of infertility in families (8). This study has been done by the aim of determining the adoption acceptance value score and effective factors placed upon in infertile couples. Thus the findings of the study may be used to better represent a way to promote adoption-friendliness in families 

## Materials and methods

227 infertile couples referred to Yazd Research and Clinical Center for Infertility of Shahid Sadoughi University of Medical Sciences between October 2009-2010 enrolled consecutively through simple random sampling in this cross-sectional study. 27 couples excluded due to refusing to participate in the study. Data were gathered by researchers through face-to-face interview and a self-designed questionnaire. 

The questionnaire consists of 25 questions classified in 4 parts. 1) Demographic variables including age, duration of marriage, educational level, occupation and location of living. 2) onus of infertility, history and the number of previous use of ART and the history of infertility in couple`s relatives. 3) View point of couples and their parents about the adoption. 4) preferred characteristics of adopted child. Questions selected from the review of the articles and expert consensus. For reliability analysis, Cronbach’s α coefficient was used to test reliability of the questionnaire and value of 0.7 or higher was considered satisfactory (9). 

The Content Validity Index for Scales (S-CVI) was also calculated for the process of content validity proposed by Polit *et al* (10). Validity was established by review of a panel of experts including four experienced psychologists. Judgments of these experts were rated on a 4-point ordinal scale with the labels of: 1) not relevant, 2) somewhat relevant, 3) quite relevant, and 4) highly relevant. It has been also indicated that an S-CVI of 0.8 or higher is acceptable (11). 

For all subscales of the questionnaire, Cronbach’s α was 0.706. The S-CVI was also computed to be 0.826. Couple’s agreement to adopt, personal views on adoption, the act of adoption itself, the consent of spouses and the consent of parents about adoption considered and scored for evaluation of adoption acceptance value score. The maximal score was 18 and the minimal was 0.

**Statistical analysis**

Descriptive statistics and statistical analysis was performed using the software package SPSS 13. Tables and indexes have been derived from the data while using ANOVA test. p<0.05 was considered statistically significant.

## Results

In this study the average age of women was 29.33±5.35 years and the average age of men was 33.69±5.82 years. Among the studied infertile couples, 44 couples (22%) were native residents to the province and 156 couples (78%) were non-native residents. 

The major cause of infertility in 69 couples (34.5%) was male factor, in 58 couples (28.8%) was female factor, in 25 couples (12.5%) was both male and female factor and in 48 couples (24.3%) there was no discernible cause. In terms of the level of education, high school and pre-university group had the greatest frequency rate including 48% men and 49.5% women. In terms of occupational status, the highest frequency rate was belonged to male with self-employment (48.5%) and female with homemaking as their job (71.5%) (Table I).

Within the study of infertile couples, 118 couples (59.2%) previously considered treatment and had used a means in order to treat their infertility. This was once for 42 couples (35%), two times, for 31 couples (27%), and three or more times for 45 couples (38%). 57 men (28.5%) and 61 women (30.5%) had the occurrence of infertility in their relatives. 28 men (14%) and 21 women (10.5%) had the experience of adoption in their relatives. (Table I). 

72 men (36%) and 87 women (43.5%) expressed their satisfaction with adoption as an alternative way of infertility treatment. 76 men (38%) and 95 women (47.5%) had considered adoption in their mind. Among 200 infertile couples under study, only 9 couples (4.5%) had adopted a child. Analysis of study population shows that the gender of adopted child was important for 42.5% of men and 43% of women. 60.7% of men preferred to adopt boy and 56.2% of women preferred to adopt a girl. 

The most favorable age for the adopting child in men and women was neonatal period. Most of men and women preferred to have information about the true parent of their adopted child. Among the studied infertile couples, most of them preferred to choose the adopted child from welfare organization. Choosing the adopted child from strangers and relatives were in next ranks (Table II). 

No significant statistical relation was found between gender, age, education level, and onus of infertility, the number of previous referrals to treat infertility and the experience of adoption in female relatives with the adoption acceptance value score. It means that these factors have no effect on the adoption acceptance value score (Table III, IV). It was found a significant statistical relation between the duration of marriage and the experience of adoption in male relatives with the adoption acceptance value score. That means the score of adoption acceptance in infertile couples rises with the increment in length of the marriage duration and the experience of adoption in male relatives (Table III).

**Table I:** Baseline characteristics of the study population

**Characteristics **			**Men (n=200)**	**Women (n=200)**
Age (year)			33.69 ± 5.82	29.33 ± 5.35
Education level				
		Illiterate	7 (3.5)	6 (3.0)
		Elementary school	15 (7.5)	18 (9.0)
		Guidance school	33 (16.5)	29 (14.5)
		High school and pre-university group	96 (48.0)	99 (49.5)
		Collegiate	49 (24.5)	48 (24.0)
Occupation status				
		Self-employed	97 (48.5)	12 (6.0)
		Worker	33 (16.5)	4 (2.0)
		Employee	63 (31.5)	28 (14.0)
		Student	4 (2.0)	12 (6.0)
		Retired and unemployed	2 (1.0)	1 (0.5)
		Homemaker	0 (0.0)	143 (71.5)
Living status				
		Native	44 (22.0) *	44 (22.0)*
		No native	156 (78.0)	156 (78.0)
Location of living				
		Center of province	86 (43.2) *	86 (43.2) *
		Other cities of province	92 (46.0)	92 (46.0)
		Village	22 (10.8)	22 (10.8)
Onus of infertility				
		Woman	58(28.8)*	58(28.8)*
		Man	69(34.5)	69(34.5)
		Both	25(12.5)	25(12.5)
		Unknown	48(24.3)	48(24.3)
Previous referrals to treat infertility			118 (59.2) *	118 (59.2)*
The number of previous referrals to treat infertility				
	One		42 (35.0) *	42 (35.0) *
	Two		31 (27.0)	31 (27.0)
	Three and more		45 (38.0)	45 (38.0)
History of infertility in relatives			57 (28.5)	61 (30.5)
History of adoption in relatives			28 (14.0)	21 (10.5)

Data are presented as mean ± SD or number (%).

* These data are related to the couples.

**Table II:** Preferred characteristics of adopted child among infertile couples

**Characteristics **				**Men (n=200)**	**Women (n=200)**
Gender of adopted child					
			Boys	82 (60.7)	60 (43.8)
			Girls	53 (39.3)	77 (56.2)
Age of adopted child					
	1-day to 1-month			133(66.5)	135 (67.5)
	2-month to 1-year			56 (28.0)	56 (28.0)
	2-year to 4-years			10 (5.0)	8 (4.0)
	5-year or more			1(0.5)	1 (0.5)
Knowledge about the real family of adopted child				122 (61)	125 (62.5)
Place to select the adopted child					
		Relatives		40 (20.0)	30 (15.0)
		Strangers		57 (28.5)	67 (33.5)
		Welfare organization		103 (51.5)	103 (51.5)

Data are presented as number (%).

**Table III:** Relation of adoption acceptance value score with the baseline indices

**Characteristics**				**Men**	**p-value**	**Women**	**p-value**
Age groups					0.104		0.053
	22-29 years			5.83 ± 4.68		5.74 ± 4.37	
	30-34 years			6.58 ± 4.64		7.06 ± 4.38	
	35-39 years			7.30 ± 5.48		7.90 ± 5.05	
	40-53 years			8.57 ± 5.85		8.71 ± 5.41	
Duration of marriage					0.002		0.004
		1-5 years		5.64 ± 4.25		6.26 ± 3.96	
		> 5 years		7.85 ± 5.53		8.20 ± 5.28	
Education level					0.082		0.354
		Illiterate		7.42 ± 6.60		9.00 ± 6.70	
		Elementary school		9.33 ± 5.47		8.00 ± 5.64	
		Guidance school		7.00 ± 5.44		8.50 ± 5.62	
		High school and pre-university group		7.05 ± 4.98		6.90 ± 4.40	
		Collegiate		5.32 ± 4.41		6.80 ± 4.43	
Onus of infertility					0.489		0.966
			Woman	7.36 ± 5.33		7.36 ± 4.77	
			Man	6.94 ± 5.47		7.36 ± 5.02	
			Both	6.92 ± 4.66		7.48 ± 4.84	
			Unknown	5.87 ± 4.40		6.97 ± 4.60	
History of adoption in relatives					0.004		0.269
			Yes	9.32 ± 5.24		8.38 ± 5.26	
			No	6.40 ± 4.95		7.15 ± 4.73	

All the parameters in this table are expressed in Mean±SD with ANOVA test.

**Table IV:** Relation of adoption acceptance value score with the number of previous referrals to treat infertility

**The number of previous referrals**	**Mean**	**p-value**
One time	7.14±4.90	0.257
Two times	7.84±4.70	
Three times and more	8.40±5.20	

The data in this table are expressed in Mean±SD with ANOVA test.

## Discussion

Infertility is one of the most contentious issues in today’s society. Nowadays artificially assisted reproductive techniques are used to cure infertility. However, these methods not only impose highly expensive treatment costs but they are also generally time-consuming (3-5). This is aside from the fact that they have low success ratio which is usually around 20-40% (3, 5). 

One of the best alternate methods for infertility treatment is to considering adoption. This is because adoption often decreases the treatment costs and the mental pressures within an infertile couple (7, 8). This study has been done with the aim of determining adoption acceptance value scores and the factors associated with the adoption acceptance. 200 couples referred to Yazd Infertility Center of Shahid Sadoughi University of Medical Sciences enrolled randomly in this study. Findings shows the average age of women in the study was 29.33±5.35 and the average age of men in the study was 33.69±5.82. There was no significant statistical relation between the gender and the adoption acceptance value score. It means that gender have no effect on the adoption acceptance value score. The results of an evaluation which have been accomplished by American Center for Control and Prevention of illnesses in the year of 2002 shows that men have adopted a child two times more than women aged between 18-44 years old (12). The different results may be due to age range and inclusion of adoptions occurred in separated and remarried parents while the present study included only infertile couples. 

In the present study, although there is no significant statistical relation between gender and the adoption acceptance value score, but tables shows that the adoption acceptance average score increases by the increment of the age of both genders and this finding is in consistence with the results of the studies accomplished in America (12-14), Pakistan (Karachi) (15) and Nigeria (16). 

There is also a direct relationship between score of adoption acceptance and duration of marriage it means that in marriage with more than 5 years duration, infertile couples are more thoughtful about adoption, a finding which is consistent with the results of the studies accomplished in America (12-14), Pakistan (Karachi) (15) and Nigeria (16).

Statistical analysis in this study reveals that there is no significant relation between education level with the rate of adoption acceptance and this is in contrary to the findings of the American Center for Control and Prevention of Illnesses studies done in 2002 and 2009 which is in favor of direct relationship between the adoption acceptance score and educational level of couples (12, 13).

Considering the onus of infertility it was female factor in 28.8% of cases, male factor in 34.5% of cases, both male and female factor in 12.5% of cases and in 24.3% of cases there was no discernable cause. These findings are in consistency with previous results which are in favor of this point that onus of infertility is female factor in 30-40% of cases, male factor in 30-40% of cases, both male and female factor in 10-20% of cases and in 15-25% there was no discernable cause (3-5).

There was no significant statistical relation between the onus of infertility and adoption acceptance value score. This finding is in contrary to the result of previous studies which indicate that among 70-80% of couples who are applicants to adopt a child, the cause of infertility is male factor (17-19). This difference may be due to this reason that in the present study, statistical population were infertile couples who considered adoption as the last option and they were still hopeful to cure their infertility. Moreover, they announced their views on adoption independently and without being aware of the other partner’s opinion. So due to the patriarchal attitudes towards adopting a child, couples whose infertility cause is male factor have more tendencies towards adoption but couples whose infertility cause is female factor may regard second marriage of men as a solution rather than adoption.

Although in the present study no significant statistical relation was found between the number of previous referrals to treat infertility and the adoption acceptance value score but the statistics show that the adoption acceptance value score average rises when the number of previous referrals for infertility cure increases. This finding is in consistency with the results of previous studies (12-14, 16) which indicate that the adoption acceptance score is higher between women who have used the infertility services.

The results reveal that there is a significant relation between the presence of adoption background in men’s relatives and the adoption acceptance value score. That means that the adoption acceptance value scores are higher between men who have had the experience of adoption in their relatives. But this relation was not found among women in the study. However, the average of adoption acceptance value score was higher in women who have had the experience of adoption in their relatives in comparison to women who have not had such experience.

According to the present study, the favorable gender for the adopted child in 60.7% of men and in 56.2 percent of women was girl. According to statistics of the Semi-family Affairs Office most of the applicants for adoption from the outset want to adopt a girl rather than a boy (6). This difference may be due to the fact that in the present study, we asked about the opinions of the men and women in the study towards their preferred gender separately. 

However, applicant couples choose the favorable gender in accordance to the agreement between theirselves and by considering different factors such as canonical issues, friendliness, special tendency and less responsibility. Although according to the National Information report on adoption and its related conducts, the acceptance rates of adopting boy and girl were the same between American married women around the age of 18-44 in 1999, but most women preferred girl rather than boy that is as same as our results (14).

According to the present study, the favorable age for the adopted child in most of the cases (95 percent) was less than one year old. This finding is in consistency with the finding of American Center for Control and Prevention of Illnesses in 2002 which indicate that most of the women like to adopt children under two years old who are healthy, without any inability, and alone without sister or brother (12).

According to the results, most of the cases want to have information about the real parents of the adopted child. This finding is in consistency with a study accomplished by Esmaili which indicate that most of the adoption applicants like to know about the biological parent of the child (20). 

According to the present study, between the infertile couples population the favorable place for choosing the adopted child was the State Welfare Organization. This was in consistency with the result of the previous studies (20, 21). However according to the research study in Pakistan (Karachi) most of the cases preferred to choose the adopted child among their relatives due to the same blood (15).

## Conclusion

In regards to highly expensive treatment costs, time-consuming and generally low success rate for most Infertility treatments, Adoption serves as an alternate option for infertile couples requires substantial reforms in society and greater acceptance from infertile couples as well.
